# Relationship among land surface temperature and LUCC, NDVI in typical karst area

**DOI:** 10.1038/s41598-017-19088-x

**Published:** 2018-01-12

**Authors:** Yuanhong Deng, Shijie Wang, Xiaoyong Bai, Yichao Tian, Luhua Wu, Jianyong Xiao, Fei Chen, Qinghuan Qian

**Affiliations:** 1 State Key Laboratory of Environmental Geochemistry, Institute of Geochemistry, Chinese Academy of Sciences, 99 Lincheng West Road, Guiyang, 550081 Guizhou Province PR China; 2 University of Chinese Academy of Sciences, Beijing, 100049 PR China; 3 Puding Karst Ecosystem Observation and Research Station, Chinese Academy of Sciences, Puding, 562100 PR China; 4 School of Geography and Environmental Sciences, Guizhou Normal University, Guiyang, 550001 Guizhou Province PR China

## Abstract

Land surface temperature (LST) can reflect the land surface water-heat exchange process comprehensively, which is considerably significant to the study of environmental change. However, research about LST in karst mountain areas with complex topography is scarce. Therefore, we retrieved the LST in a karst mountain area from Landsat 8 data and explored its relationships with LUCC and NDVI. The results showed that LST of the study area was noticeably affected by altitude and underlying surface type. In summer, abnormal high-temperature zones were observed in the study area, perhaps due to karst rocky desertification. LSTs among different land use types significantly differed with the highest in construction land and the lowest in woodland. The spatial distributions of NDVI and LST exhibited opposite patterns. Under the spatial combination of different land use types, the LST–NDVI feature space showed an obtuse-angled triangle shape and showed a negative linear correlation after removing water body data. In summary, the LST can be retrieved well by the atmospheric correction model from Landsat 8 data. Moreover, the LST of the karst mountain area is controlled by altitude, underlying surface type and aspect. This study provides a reference for land use planning, ecological environment restoration in karst areas.

## Introduction

Land surface temperature (LST) is a significant parameter in exploring the exchange of surface matter, surface energy balance and surface physical and chemical processes and is currently widely used in soil, hydrology, biology and geochemistry^1,2^. Land use/cover change (LUCC) is an important factor affecting LST. The surface reflectance and roughness of different land use types are different, thereby leading to differences in LST^3^. Furthermore, in the context of urbanisation, the intensity of human activities is enhanced and the surface cover is rapidly changed^4^. Therefore, the relationship between LST and LUCC should be investigated to further analyse the ecological effects of LST and address regional environmental problems. Vegetation can effectively influence LST by selectively absorbing and reflecting solar radiation energy and regulating latent and sensible heat exchange^5^. Normalised difference vegetation index (NDVI) is a vegetation indicator that is generally utilised in the study of the relationship between LST and vegetation^6–8^. Because the relationship of LST and NDVI, affected by many factors, is quite complex^9–11^, it is necessary to further explore the relationship between LST and NDVI.

Since the 1970s, domestic and foreign scholars have been proposing mature methods that are based on thermal infrared remote sensing data for retrieving LST^12^. Currently, many scholars apply remote sensing to analyse the relationship among LST, land use and NDVI^9,13,14^. However, some problems and shortcomings in the existing research still need to be addressed. Different research methods, such as directly using brightness temperature^15^, have been utilised to determine LST, thus reducing data accuracy. Furthermore, in most previous studies, the main study areas were large cities, such as Tokyo^16^, Shanghai^17^, Bangkok^18^, and the main research content was the effect of urbanisation-related land changes and urban heat island effect in these cities on LST^19^. Only a few studies have been conducted about the relationship among LST, LUCC and NDVI in karst areas characterised by unique geographical features and fragile ecological environment^20,21^, particularly in economically poor karst areas with serious rocky desertification caused by soil erosion^22,23^. LST is an important factor to reflect the environmental changes of the underlying surface and the physical and chemical processes in karst area. Thus, it is necessary to further explore the influence mechanism of LST and LUCC, NDVI in karst area.

To reveal the characteristics of land surface temperature in karst area, we selected a typical karst area, i.e. the Yinjiang County of Guizhou Province, China, as study area and calculated the LST and NDVI from Landsat 8 remote sensing images to address the above mentioned issues. Meanwhile, the land use type map was obtained by CART decision tree classification. This study aimed (1) to analyse the inversion accuracy and spatial distribution characteristics of the LST in the study area, (2) examine the relationship between LST and its influencing factors in the karst mountain area, (3) explore the relationship between LST and different land use types and (4) determine the relationship between LST and NDVI.

## Results

### LST inversion results

Due to the lack of measured LST for verifying the inversion accuracy, the meteorological site temperature data were used for validation, which showed that the lowest, highest and average temperatures of Yinjiang County were 19 °C, 29 °C and 24 °C, respectively. The LST calculated by the atmospheric correction method was 14.75 °C to 40.59 °C, and the mean LST was 27.67 °C. Furthermore, the statistics showed that the LST of 99% of the study area was within 14.75 °C to 32.40 °C, thereby indicating that the LST retrieved by the atmospheric correction method was the same as the actual LST.

**Figure 1:**
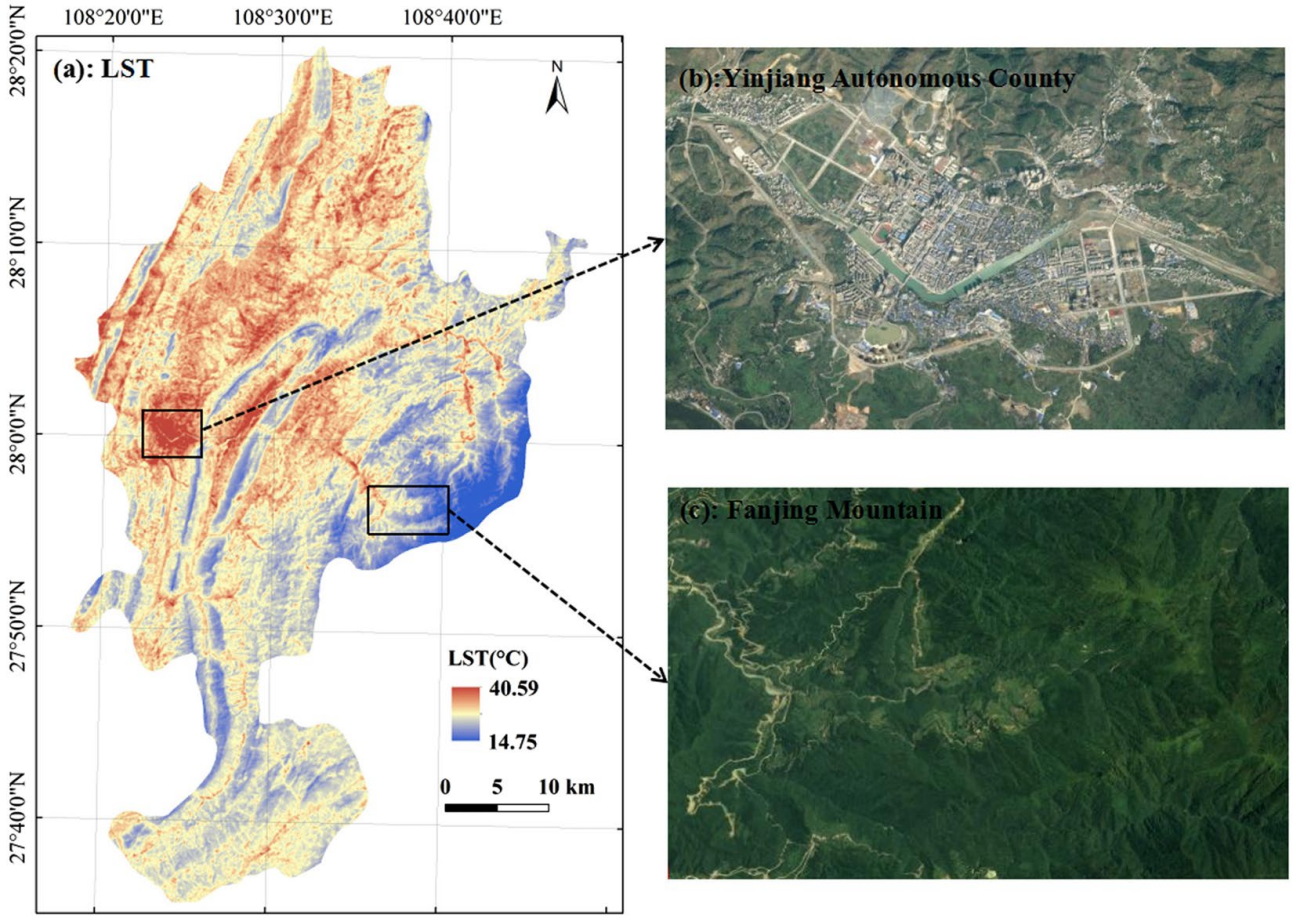
Land surface temperature (LST) of the study area (**a**); the high-definition satellite maps of the county (**b**) and Fanjing Mountain (**c**). Figure 1a was generated though the ArcGIS 9.3 software (http://www.esri.com). Figures 1b and c were obtained by SimpleGIS 2.7.1 (http://www.rscloudmart.com/application/120173.htm).

In Fig. 1a, the LST of the study area was high in the west and north and low in the east and south and showed an overall downward trend from northwest to southeast. Moreover, the LST formed a high-value area in Yinjiang County and other construction land-intensive areas, thus showing a regular urban heat island phenomenon (Fig. 1b). In the east of the study area, i.e. Fanjing Mountain, the LST formed a low-value area (Fig. 1c). The combination of the study area DEM elevation and land use data was used for superposition analysis, showing that the trend was mainly due to the combined effects of land use and topography. In addition, the combination of high-definition satellite maps showed high-temperature zones different from the traditional urban heat island in the county suburbs. Moreover, the highest LST was observed in the suburbs of the county.

### Relationship between LST and elevation

**Figure 2:**
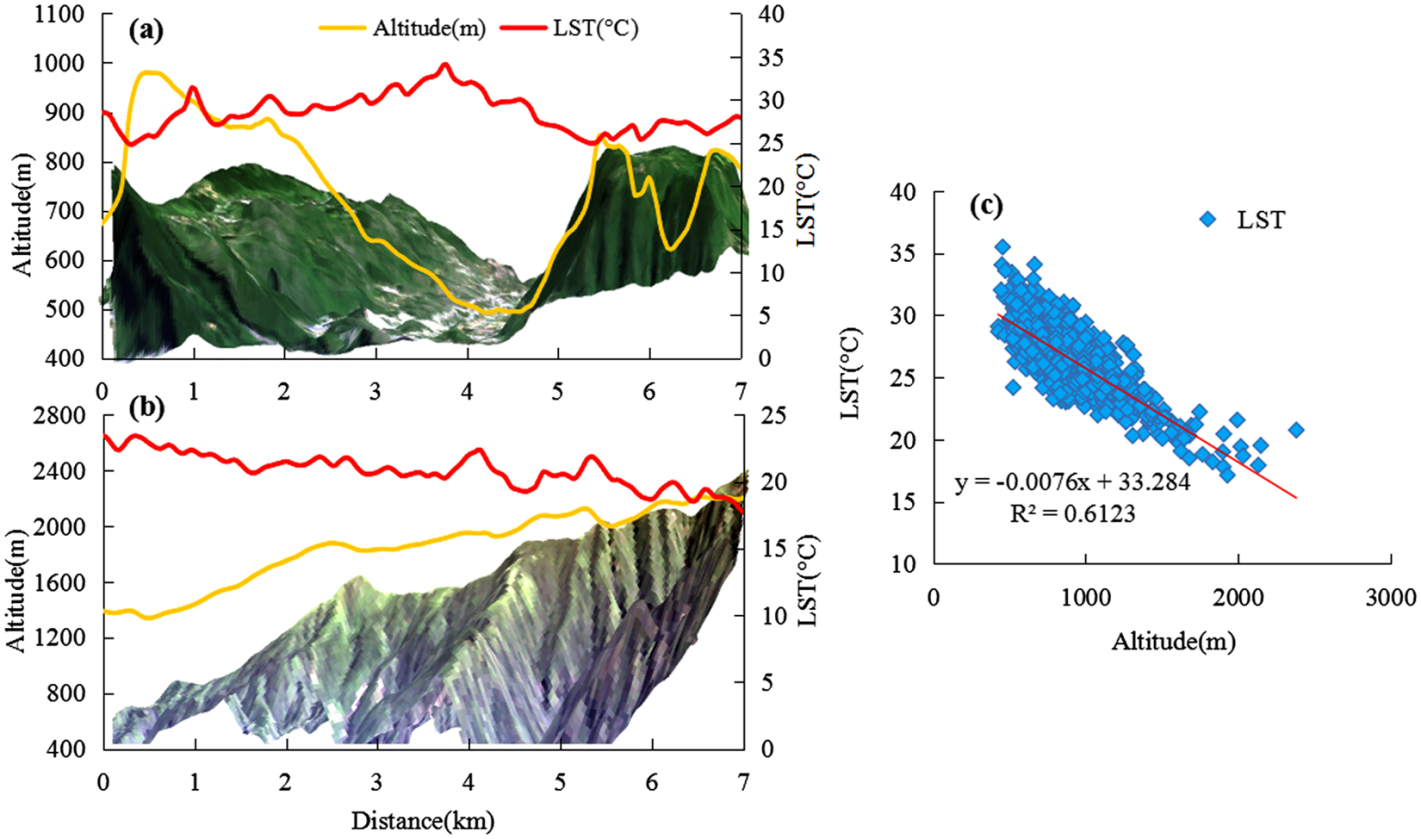
The land surface temperature (LST) profiles and altitude profiles of Langxi Valley (**a**) and Fanjing Mountain (**b**); the scatter plot between LST and altitude (**c**). Figures 2a and b were generated through ArcGIS 9.3 (http://www.esri.com). In Fig. 2c, SPSS 22.0 (https://www.ibm.com/analytics/cn/zh/technology/spss/spss-trials.html) was used to perform regression analysis on the LST and altitude.

The spatial distribution of the LST and altitude exhibited an opposite pattern. As shown in Supplementary Fig. S1, the LST colour changed from red to green from northwest to southeast, i.e. the LST gradually decreased with the increase in altitude. In the west–east and northwest–southeast directions, the terrain distribution alternated between valleys and hills. Correspondingly, the high-value peaks and low-value valleys of the LST were alternately distributed. Furthermore, in the area with relatively high elevation difference, the LST had a noticeably vertical variation rule with altitude change. Taking Fanjing Mountain and Langxi Valley as examples, Fig. 2a shows a low altitude in Langxi Valley area, where the LST was high. Figure 2b shows that the LST of Fanjing Mountain gradually decreased with the increase in altitude. However, the profile curves of the LST fluctuated more frequently than that of the corresponding elevation profiles. Meanwhile, in Fig. 2c, LST had a significant negative correlation with altitude, with a correlation coefficient of −0.782. The drop rate of the LST was 7.6 °C/km, which is 1.26 times that of the global average (6.5 °C/km). It is mostly probably because the underlying surface of the study area is complex, such as the hot valley, which is quite different from the area where the underlying surface is homogeneous.

### Relationship between LST and land use

#### LST characteristics of different land use types

LSTs among different land use types were ranked as follows: construction land > unused land > cultivated land > water body > grassland > woodland (Table 1). The LST of construction land and its standard deviation were the highest. Overall, the LST of construction land, unused land and cultivated land, which had high human activity intensity, was high; the LST of water body, forest land and grassland, which had low human activity intensity, was low. Therefore, land use types should be rationally planned and a cooling effect should be induced through green vegetation and water.

**Table 1:** The mean LST and its standard deviation in different land use/cover type, which were calculated by means of GIS spatial partition statistics.

Class	Water	Woodland	Construction land	Cultivated land	Grassland	Unused land
LST(°C)	27.14	25.04	32.25	27.92	26.51	27.89
Std. deviation	1.18	1.50	2.63	1.45	1.37	1.96

Table 2 shows the post hoc test results of the mean differences in the LST among the different land use types in the study area^11^. The table has a 6 × 6 symmetrical matrix with 15 combinations. Except for that between the cultivated and unused lands, the differences among the pairs (14 pairs) were significant (sig. <0.01). The cultivated land was mainly dry land with low vegetation coverage, whereas the unused land was mainly bare rock in karst and sparsely vegetated grassland. Thus, the cultivated and unused lands exhibited only a slight difference in surface emissivity, thereby resulting in LSTs with no significant difference. Influenced by different heating capacity, heat conduction and thermal radiation properties, the LSTs of the woodland, grassland, water body and construction land exhibited significant differences, indicating that the different land use types contributed differently to the regional thermal environment effect.

**Table 2:** Multiple comparison table of mean differences in LST (°C) for different surface types.

Class	Water body	Woodland	Construction land	Cultivated land	Grassland	Unused land
Water body		2.09^*^	−5.86^*^	−0.78^*^	0.63^*^	−0.85^*^
Woodland	−2.09^*^		−7.22^*^	−2.88^*^	−1.47^*^	−2.94^*^
Construction land	5.86^*^	7.22^*^		4.34^*^	5.75	4.28^*^
Cultivated land	0.78^*^	2.88^*^	−4.34^*^		1.41^*^	−0.07
Grassland	−0.63^*^	1.47^*^	−5.75^*^	−1.41^*^		−1.47^*^
Unused land	0.85^*^	2.94^*^	−4.28^*^	0.07	1.47^*^	

^*^Significant correlation at the 0.01 level (bilateral).

#### LST features of different land use types based on slope direction and elevation

The solar radiation received by the earth is different because of the difference in slope directions of the earth’s surface, which leads to the difference in LST^24^. In this study, the imaging time for Beijing was 11:15:48 a.m. and the mountain direction of the study area was southeast for the sunny slope and northwest for the shady slope. In the study, the mean LST of the sunny slope was 1.92 °C higher than that of the shady slope. In addition, in Supplementary Fig. S2, the average LST of each land use type per 100 m altitude range was obtained. Overall, in each altitude interval, namely at approximately the same altitude, the LST of the different land use types exhibited differences, and their performances were ranked as follows: unused land > cultivated land > construction land > grassland > water body > woodland. Meanwhile, the range of LST change varied across different classes and altitudes. In addition, for all land use types, LST was negatively correlated with the elevation, and the linear goodness of fit (*R*^2^) was more than 0.9, excluding that of unused land.

As shown in Table 3, under sunny slope conditions at the same altitude, the LSTs of all land use types were ranked as follows: unused and construction lands > cultivated land > grassland > forestland > water body. By contrast, under shady slope conditions at the same altitude, the LSTs of all land use types were ranked as follows: unused land > construction land > cultivated land > grassland > water body > woodland. Moreover, for each land use class in the same altitude range, the LST under sunny slope conditions was higher than that under shady slope conditions. The maximum difference could reach 5.14 °C (Supplementary Table S1). Water body was excluded because the water bodies in lowlands, such as valleys, exhibited no noticeable difference between sunny and shady slope conditions.

**Table 3:** The LST of various land use types in shady/sunny slope under elevation per 100 m range.

Elevation (m)	Sunny slope (°C)						Shady slope (°C)					
	Water body	Woodland	Construction land	Cultivated land	Grass land	Unused land	Water body	Woodland	Construction land	Cultivated land	Grassland	Unused land
400–500	28.62	29.31	29.85	30.69	29.56	32.31	28.16	28.28	30.07	29.47	29.8	31.18
500–600	28.56	28.81	29.51	30.53	29.36	32.47	28.18	28.17	29.31	29.14	28.87	28.99
600–700	27.69	28.31	29.57	29.99	28.82	34.33	27.79	27.62	28.43	28.46	28.16	29.20
700–800	27.25	27.79	29.23	29.42	28.39	32.95	27.65	27.03	28.51	27.85	27.68	29.00
800–900	26.61	27.08	30.06	28.89	27.94	30.63	26.52	26.22	28.20	27.19	26.97	28.50
900–1000	26.13	26.35	29.80	28.44	27.52	28.73	26.22	25.68	27.23	26.63	26.62	27.58
1000–1100	25.97	25.72	28.84	27.54	27.06	29.03	25.35	25.01	27.97	26.04	26.02	31.11
1100–800	25.04	25.25	29.80	26.94	26.36	27.28	25.37	24.51	27.57	25.65	25.65	25.95
800–1300	24.41	25.08	29.32	26.79	25.95	29.50	25.08	24.45	—	25.53	25.24	27.20
1300–1400	—	24.36	28.16	25.90	25.58	27.50	—	23.45	—	25.01	25.07	24.90
1400–1500	—	23.44	29.63	25.58	25.34	26.76	—	22.58	—	24.85	25.91	26.40
1500–1600	—	22.55	—	24.40	23.90	—	—	22.07	—	24.10	—	—
1600–1700	—	21.45	—	—	21.54	—	—	21.03	—	—	—	—
1700–1800	—	21.22	—	—	—	—	—	20.55	—	—	—	—
1800–1900	—	20.52	—	—	—	—	—	19.91	—	—	—	—
1900–2000	—	20.51	—	—	—	—	—	19.21	—	—	—	—
2000–2100	—	20.09	—	—	—	—	—	19.22	—	—	—	—
2100–2200	—	19.87	—	—	—	—	—	19.34	—	—	—	—
2200–2300	—	19.50	—	—	—	—	—	19.40	—	—	—	—
2300–2400	—	18.96	—	—	—	—	—	16.21	—	—	—	—
2400–2500	—	17.98	—	—	—	—	—	18.91	—	—	—	—

The LSTs of the various land use types were extracted from the sunny slope (southeast) and the shady slope (northwest) per 100 m elevation by using ArcGIS 9.3 software (http://www.esri.com).

### Relationship between LST and NDVI

#### Spatial distribution characteristics of LST and NDVI

**Figure 3:**
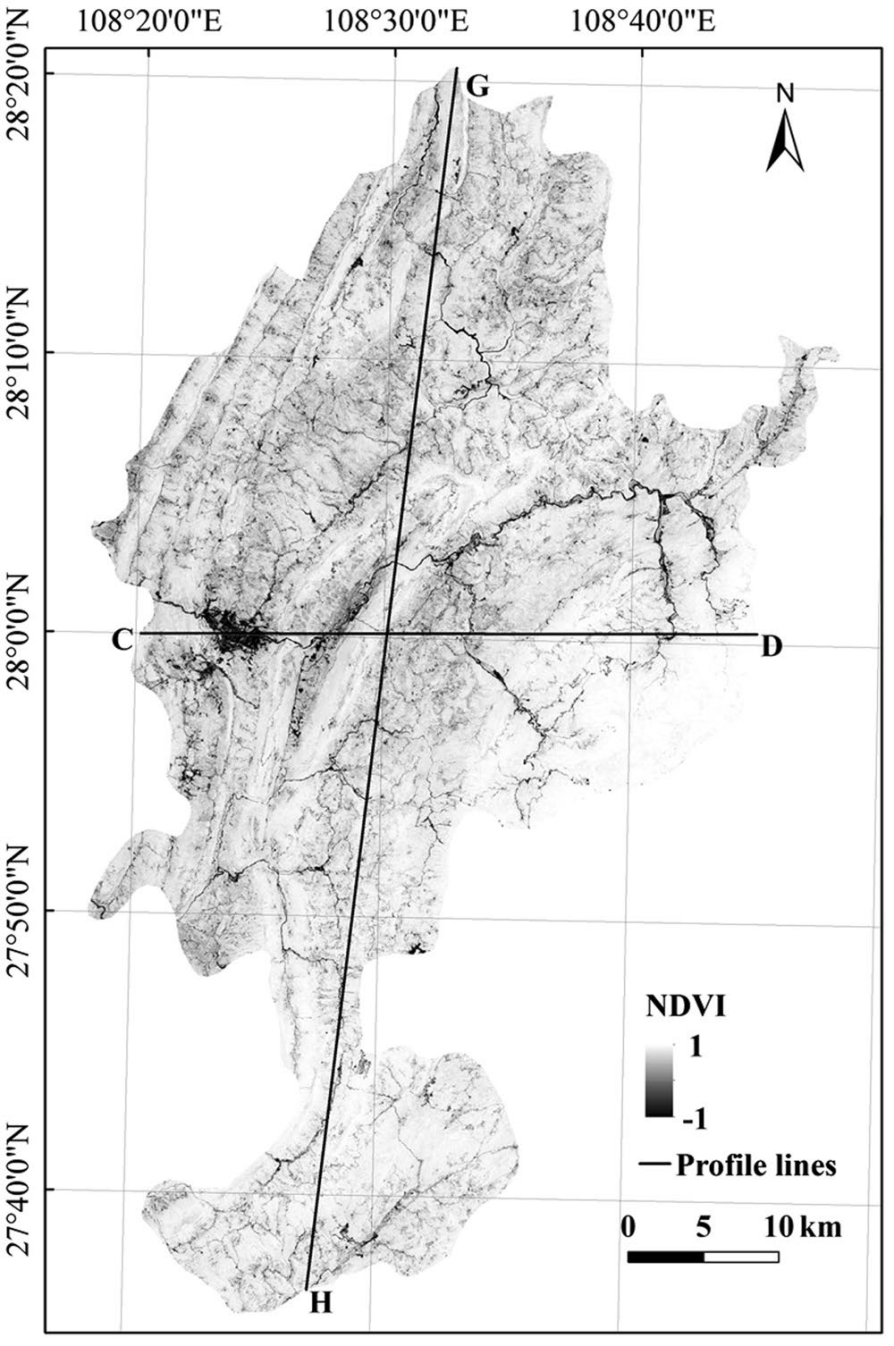
NDVI map of the study area. The image was generated by using ArcGIS 9.3 (http://www.esri.com).

**Figure 4:**
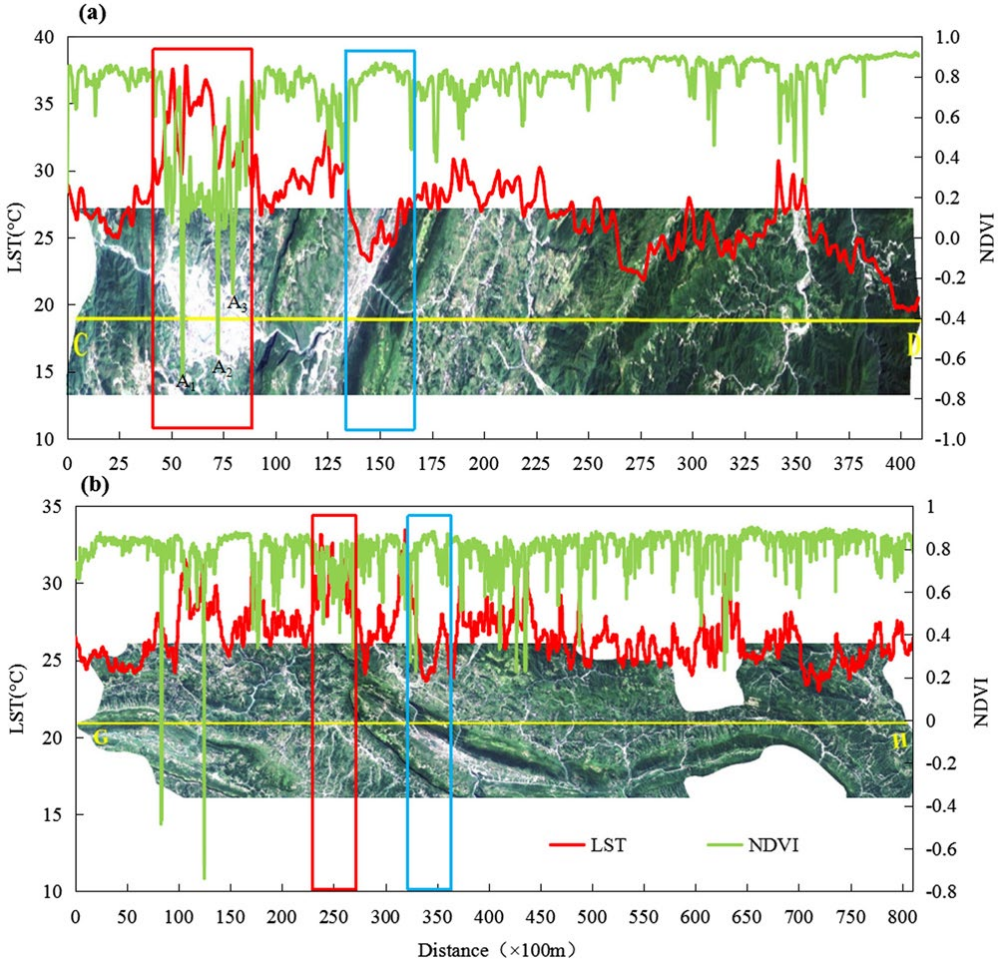
CD (west–east) (**a**) and GH (northeast–southwest) (**b**) profile maps. The LST and NDVI data were extracted from Landsat 8 data by using ArcGIS 9.3 (http://www.esri.com). The Landsat 8 images in Fig. 4 were provided by USGS^41^ (http://glovis.usgs.gov/).

Figures 1 and 3 show that the LST and NDVI exhibited opposite spatial distribution patterns. In Fig. 4a and b, the LST showed the opposite trend with the corresponding NDVI in two different directions. At the macro level, the LST in Yinjiang County formed a high-value area, thus showing the city heat island phenomenon; moreover, the low-value zones of NDVI in this area exhibited a small effect, which are marked by red boxes in Fig. 4a and b. At the micro level, the high peaks of the LST value corresponded to a low ‘valley’ of the NDVI; meanwhile, the areas marked by blue boxes show the high peaks of the NDVI that exactly corresponded to the low ‘valley’ of the LST. Three points in the CD profile, i.e. A_1_, A_2_ and A_3_, were characterised by relatively low NDVI and LST. The high-definition satellite map shows that these special points were in the Yinjiang River. The low LST and NDVI of the water body indicate its good cooling effect. The water temperature was relatively stable with vegetation coverage that was approximately zero, and the water LST was positively correlated with NDVI^25,26^. Meanwhile, the main part of the unused land was a non-vegetation area. Thus, the water bodies and unused land were not considered in the discussion about the relationship between NDVI and LST among the different land use types.

#### Quantitative relationship between LST and NDVI in single land use type

In the regression function shown in Table 4, *Y* and *X* denotes LST and NDVI, respectively, and the saliency coefficients are also listed. The table shows that the LSTs of the different land use types have negative linear relationship with the NDVI. In the regression functions, the absolute values of the first-term coefficients were ranked as follows: woodland > grassland > construction land > cultivated land, indicating that the LST of the woodland decreased fastest with the increase in NDVI, followed by grassland and construction land, and the LST of the cultivated land decreased relatively slowly. Therefore, the LSTs of the grassland and woodland were affected by vegetation cover more than the cultivated and construction lands, and their cooling effect was more noticeable than that of the other land use types. Thus, in urban lands, appropriately increasing the amount of green area in parks is conducive to improving the city thermal environment.

**Table 4:** Regression analysis results of LST and NDVI based on land use type.

Land use types	Regression function	Sample number	R^2^
Woodland	y = −30.796x + 51.279	220	0.382
Construction land	y = −10.513x+ 34.186	325	0.2058
Cultivated land	y = −8.0793x + 32.955	205	0.2688
Grassland	y = −11.751x + 35.63	180	0.135

Linear regression analysis was conducted on the LST and NDVI of each land use type, which was generated by SPSS 22.0.

#### Relationship between spatial structure of land use types and LST

**Figure 5:**
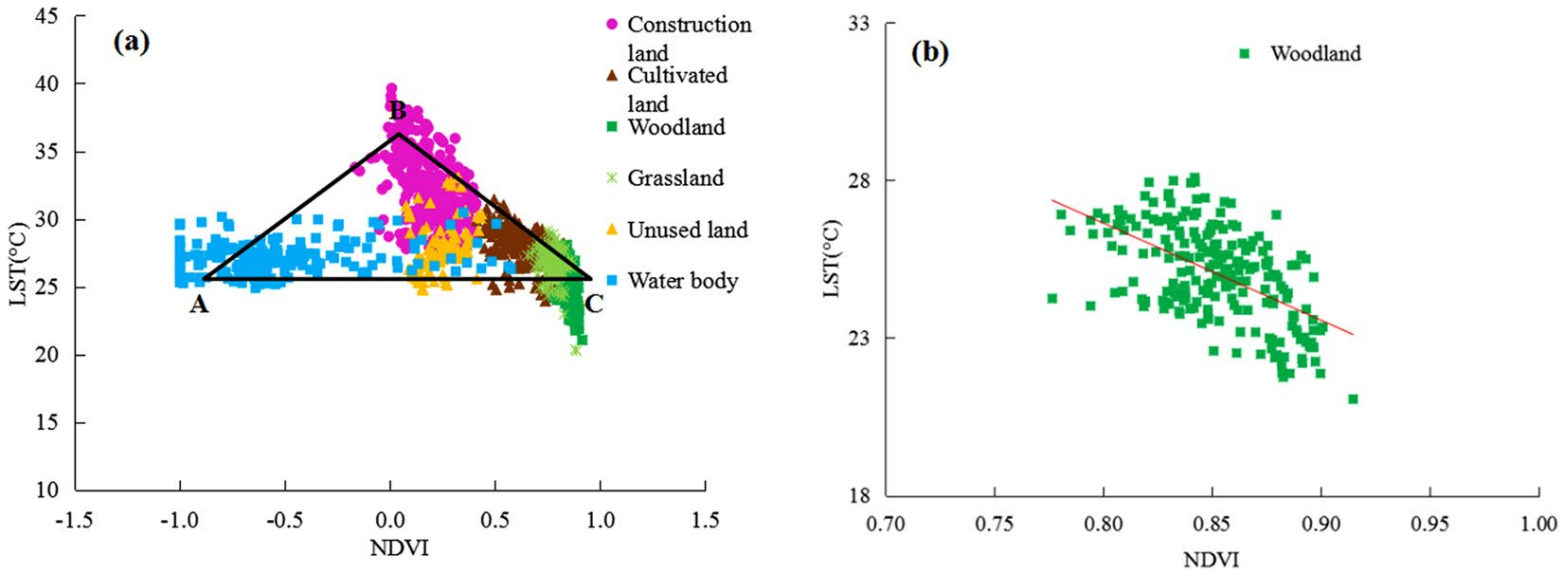
LST–NDVI feature space (**a**) and scatter diagram of LST and NDVI of woodland (**b**). The LST and NDVI data in Fig. 5a and b were extracted from LST and NDVI images randomly by using ArcGIS 9.3 (http://www.esri.com).

Because single land use is rarely observed, the relationship between LST and NDVI should be analysed in the spatial combination of different land use types. In Fig. 5a, the LST–NDVI feature space shows a unique obtuse-angled triangle shape ABC. Concretely, A denotes water body, which had low LST and approximately negative NDVI; B represents dry and bare soils (mainly construction land), which had high LST and low NDVI; and C denotes woodland, which had the lowest LST and highest corresponding NDVI. The area ratios of the six land use types constituting the feature space of the study area was ranked as follows: woodland > farmland > grassland > construction land > water body > unused land. In Fig. 5a, the different types intervened with one another in the LST–NDVI feature space; the construction land, mainly mixed with unused land, had high evaporation and low water content; the grassland was distributed in the transition zone between the cultivated land and the woodland, and its vegetation coverage was high, soil moisture was relatively abundant and evapotranspiration was small. The main distribution of the woodland was relatively concentrated and followed an overall northwest–southeast direction (Fig. 5b).

## Discussion

### Abnormal high-temperature zones

**Figure 6:**
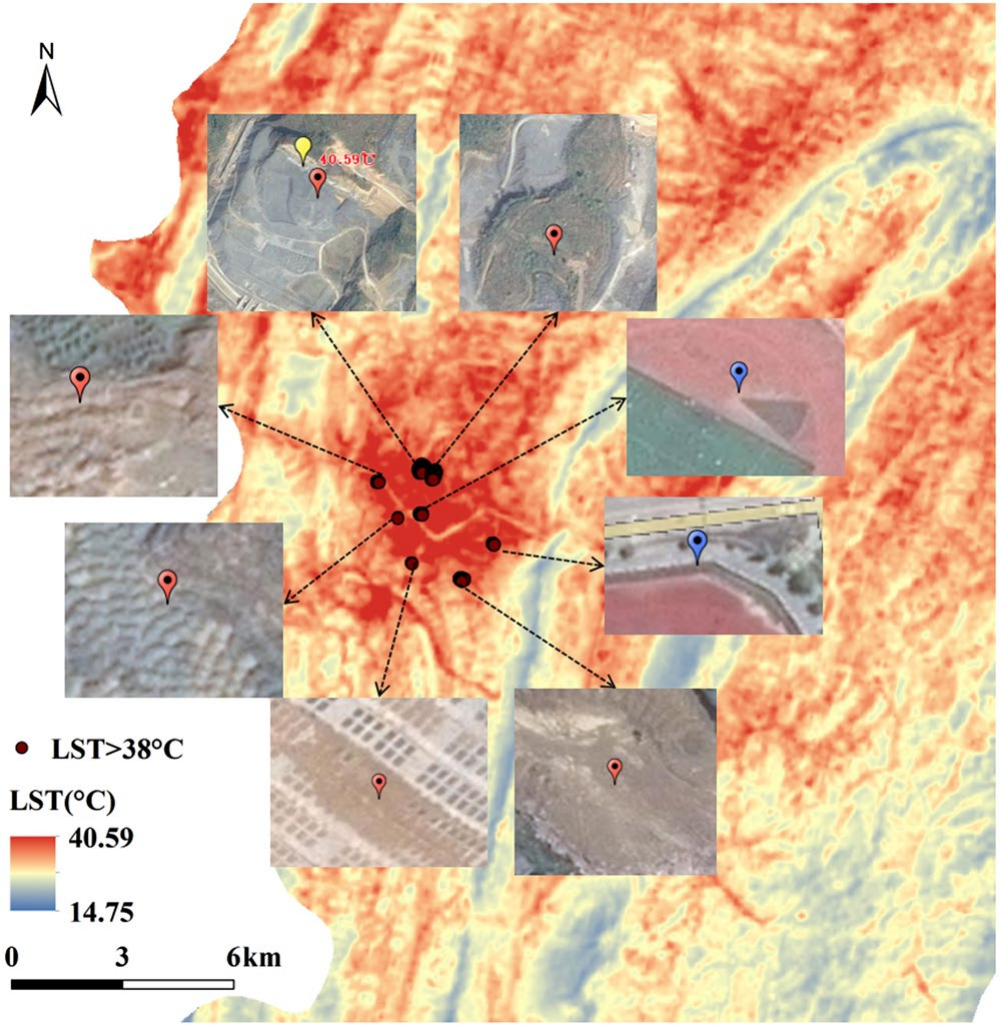
Zones of LST greater than 38 °C in the study area. The points of LST greater than 38 °C and the LST map were obtained by ArcGIS 9.3 software (http://www.esri.com). The other eight maps were the high-definition satellite images, which were generated by SimpleGIS 2.7.1 (http://www.rscloudmart.com/application/120173.htm). The blue symbols represent a conventional high temperature zone; the red symbols are marked as an abnormal high temperature zone, whereas the yellow symbol represents the highest LST of 40.59 °C in the study area.

In summer, the urban heat island effect of urban construction and industrial lands was remarkable in the study area. However, in addition to those caused by the conventional urban heat island effect, many high-temperature zones with different shapes and sizes were observed in the suburbs of the county in the high-definition satellite map. No construction was observed, and the highest LST (40.59 °C) of the study area was found there. The abnormal high-temperature zones were distributed mainly in the urban suburbs without vegetation cover and with exposed rocks (Fig. 6). In terms of the special geological environment of the study area, the karst area had a vulnerable calcium environment^27^. Barren and shallow soil leads to low vegetation coverage^28^, and the infiltration of surface water, water loss^29,30^, human disturbance and destruction cause serious soil erosion^31^, thus resulting in the exposure of bare rocks and rocky desertification^32,33^. Therefore, the specific heat capacity of the surface substances and the thermal inertia were small. Eventually, the LST of the area increased rapidly in the daytime and was higher than that in the surrounding region, thereby forming an abnormal high-temperature zone. Thus, LST of the karst area were featured not only by the conventional urban heat island effect but also by the abnormal high-temperature caused by karst rocky desertification. In addition, LST can characterise rocky desertification in karst areas to a certain extent. In future research, we can use LST as an important factor and index for analysing and evaluating the extent of rocky desertification.

### Main factors affecting the LST of the karst mountain area

Yinjiang County has prominent mountain features with mountain accounting for 76% of the county. In Supplementary Fig. S1, the terrain tilts from southeast to northwest, whereas the LST decreases from northwest to southeast and extends along the mountain range, thereby indicating a controlling role played by altitude in the LST of the study area. And the quantitative relationship shows a negative correlation between altitude and LST. This pattern is caused by two main factors. (1) The LST affected by air temperature also decreases with elevation. (2) In karst mountain areas, the surface vegetation is relatively good. Thus, the solar radiation received by the ground surface mostly spreads in latent heat form, and the LST is low. Meanwhile, the intensity and length of solar radiation differed among different slope directions. In this study, the LST also differed among different slope directions, which is consistent with the results of Wen L. J.^14^. Different land use types have different thermal capacity, thermal conductivity, roughness and surface albedo, thus leading to differences in the LST. In the study, at the same altitude in the same slope direction, the LSTs of the different land use types still differed. In summary, the LST in this study area is influenced by levels of elevation, slope direction and land use type, among which altitude plays a fundamental controlling role in the overall pattern of LST.

### LST–NDVI feature space

The existing research results on the relationship between LST and NDVI vary considerably under combinations of different underlying surface types (Table 5).

**Table 5:** Relationships between LST and NDVI.

The main author	Data	Study area	Results
Cao *et al*.^42^	Landsat ETM^+^ data on July 3, 2001	Shanghai, China	There was a nonlinear relationship between LST and NDVI in Shanghai, but the positive value of LST and NDVI showed a significant linear relationship
Hager *et al*.^43^	AVHRR data from 1981 to 1999	Mongolia	In high latitudes, LST and NDVI were positively related
Ghobadi *et al*.^9^ (2013)	Landsat 5 data in 1998 and Landsat 7 data in 2002	the South Carle Sea watershed of Iran	There was a negative correlation between LST and NDVI
Zhou *et al*.^10^	Landsat TM data on August 27, 2010	Shenyang, China	LST and NDVI scatter plots showed “triangle” relation, the three directions represent water area, green land and cultivated land, construction land, respectively
Qu *et al*.^11^	MODIS data in 2011	Shiyang River Basin in Gansu, China	LST-NDVI scatter plots showed a trapezoid distribution
Liang *et al*.^44^	Landsat TM data in 2006	Guilin, China	NDVI and LST showed a negative correlation
This paper	Landsat 8 data on August 29, 2016	Yinjiang County of Guizhou, China	LST and NDVI scatter plots showed an “obtuse-angled triangle” distribution

**Figure 7:**
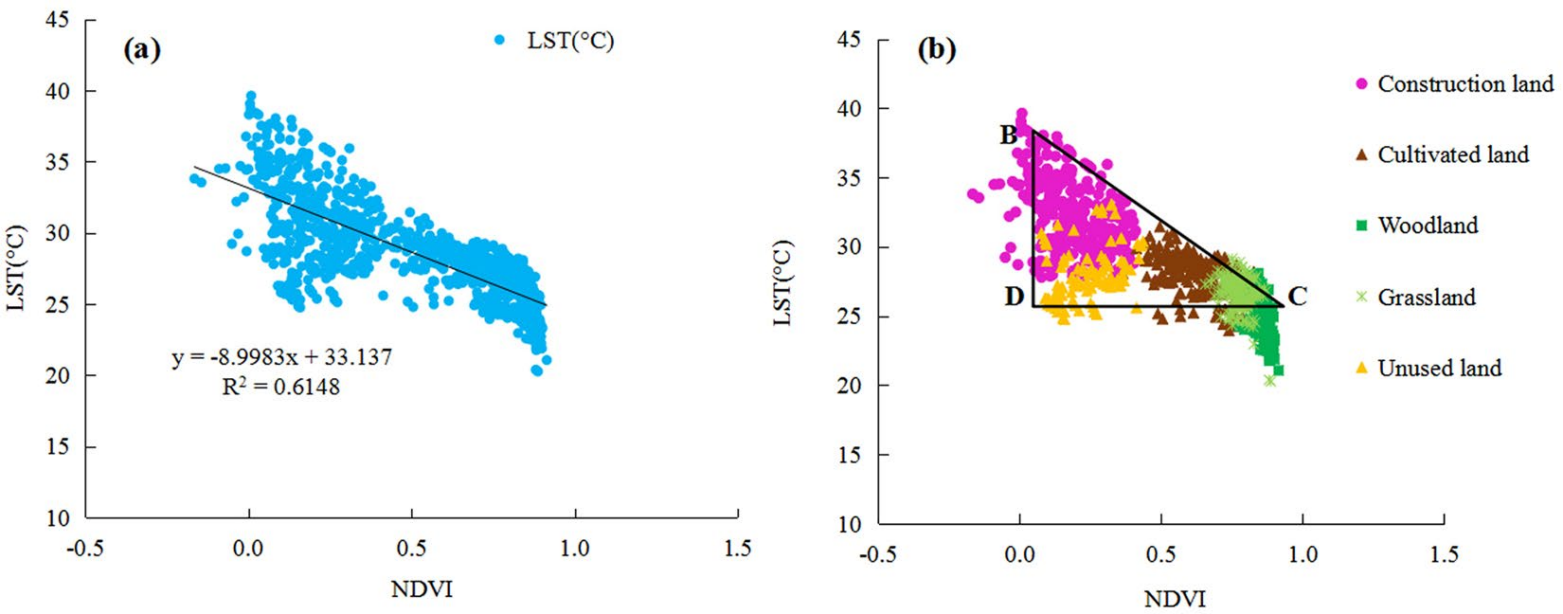
The regression analysis of LST–NDVI (**a**) and their feature space (**b**) after the removal of water body. The LST and NDVI data in Fig. 7a and b were extracted from LST and NDVI images randomly by using ArcGIS 9.3 (http://www.esri.com).

In this study, the LST and NDVI scatter diagram exhibited an obtuse-angled triangle distribution under combinations of different land use types, which is consistent with the results of Zhou Y. but different from results of other researchers. This finding could be attributed to the differences in the research methods or that between qualitative and quantitative analyses or the different soil backgrounds and sensor angles in the study area. In addition, numerous studies show that LST is positively correlated with NDVI in water bodies. Therefore, to verify whether the water caused the difference, data from water body sampling sites were removed (Fig. 7). After the removal of data from water bodies, the LST and NDVI exhibited a negative linear correlation (Fig. 7a), which is consistent with the results of Cao L. and others. Meanwhile, the LST–NDVI feature space formed a new triangle BDC (Fig. 7b), which is consistent with the findings of Price. The point D represents saturated bare soil with low LST and NDVI, and the land use type was less in this area than in the others. Meanwhile, BD represents bare edge with low vegetation coverage, whereas BC represents dry edge, which is mainly reflected in urban bare soil and dry land types^34^.

## Conclusion

In this study, the atmospheric correction algorithm was used to retrieve LST from Landsat 8 data and the LST spatial distribution patterns and influencing factors in a karst area were summarised by combining land use types with elevation data. Then, the differences in the LST among different land use types were determined, and the spatial distribution characteristics of the LST and NDVI in the study area and their quantitative relationship were discussed. The main conclusions were as follows. (1) The LST retrieved by atmospheric correction from Landsat 8 data was consistent with the actual temperature. Influenced by topography and underlying surface types, the LST of the study area showed an overall downward trend from northwest to southeast. In summer, the LST of the study area had abnormal high-temperature zones, perhaps caused by karst rocky desertification. (2) Multiple comparative analyses indicated that the LST difference among most of the land use types was significant, with construction land having the highest LST of 32.25 °C and forest land having the lowest LST of 25.04 °C. (3) For the single land use type, the water body had low LST and NDVI, while the LST and NDVI of forest land, grassland, cultivated land and construction land exhibited a negative linear correlation. (4) For the spatial structure of land use in the entire study area, the LST–NDVI feature space was an obtuse-angled triangle. After the removal of water body data, a significant negative linear relationship between LST and NDVI was observed in the quantitative analysis.

## Materials and Methods

### Study area

**Figure 8:**
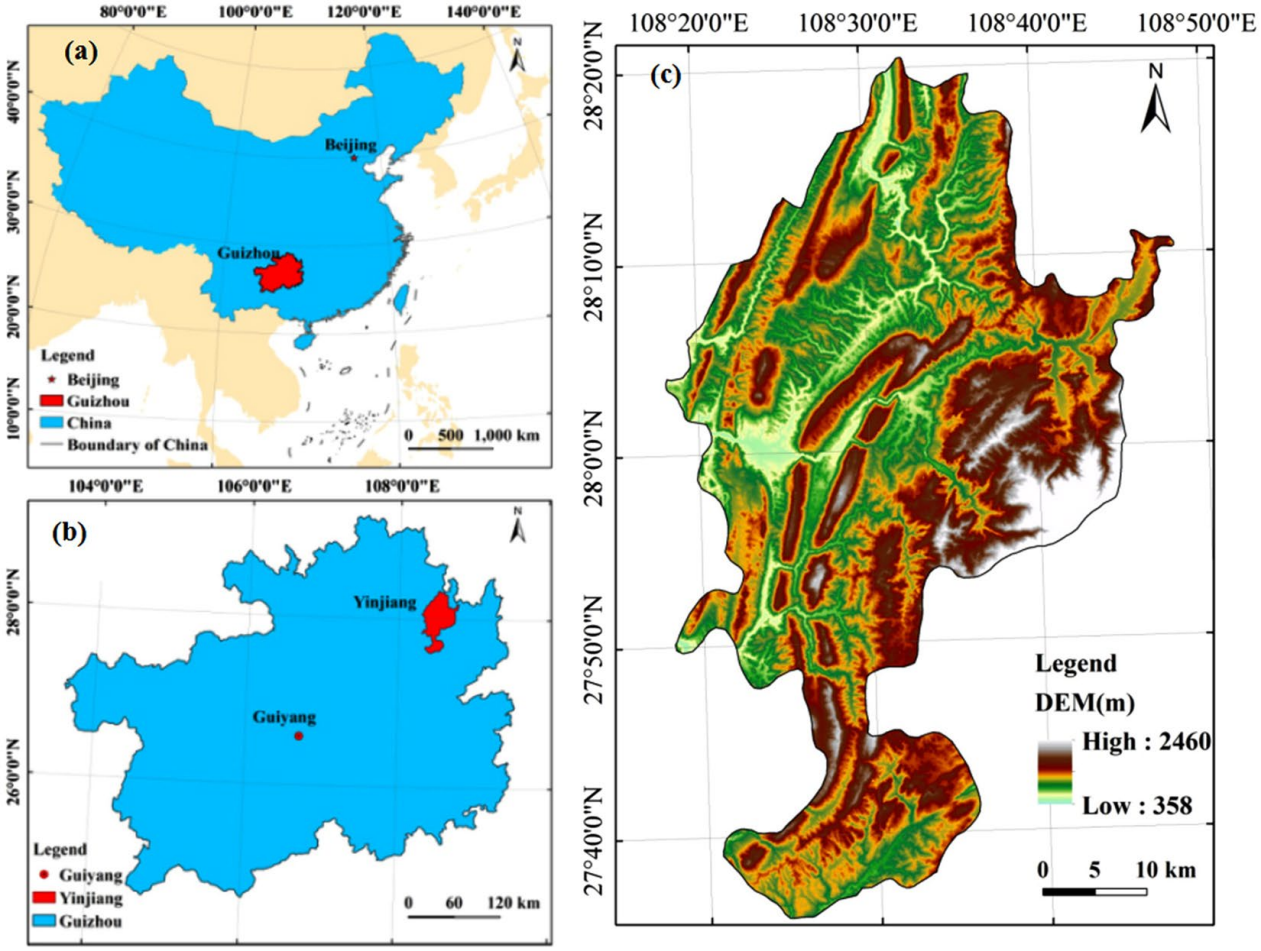
Location maps of the study area. Map of China (**a**), location of Guizhou (**b**) and DEM image of the study area (**c**). Figure 8a, b and c were generated by ArcGIS 9.3 software (http://www.esri.com). The DEM data was provided by Geospatial Data Cloud site (http://www.gscloud.cn).

Yinjiang Autonomous County is in the northeastern part of Guizhou Province in China, west of Tongren area (Fig. 8), and its geographical coordinates are 108°17′52″–108°48′18″ E, 27°35′19″–28°20′32″ N. Yinjiang Autonomous County has a land area of 1,969 km^2^ and a population of 445,000 people. Located in the northeastern area of Guizhou Plateau, Fanjing Mountain, the main peak of Wuling mountains, stands in the east of the county. In the middle are King Pier, Liangzi Slope, Eling Off and other mountains, back-like protruding terrain from south to north, such that Yinjiang County is high in the east and south, convex in the middle and low in the west and north, and its relative elevation is relatively large (Fig. 8c). The landform type is mainly karst landform, and the terrain is broken. Yinjiang County has a subtropical warm and humid monsoon climate. The average annual temperature is 15 °C to 16.8 °C, and the annual precipitation is 1,057 mm to 1,258 mm. The main vegetation types are subtropical evergreen broad-leaved forest, secondary coniferous and broad-leaved mixed forest and plantation. In addition, the soil erosion, karst, and rocky desertification areas account for 69.75%, 51.8% and 20.70%, respectively, of the study area.

### Materials

The data used in this study include Landsat 8 data, Yinjiang County Administrative Boundary, DEM data and the meteorological observation data, The Landsat 8 remote sensing images were acquired in summer, specifically on August 29, 2016, and were used for inverting the LST and calculating the NDVI. The imgaes are from the US Geological Survey and have multispectral bands with 30 m spatial resolution and a WGS84 coordinate. Without clouds over the study area, the image quality is good and the data product Level 1 T, having been geometrically corrected on the basis of terrain, can be used directly. The concrete data sources are shown in Table 6 and the technical flow chart can be found in Supplementary Fig. S3.

**Table 6:** Major data sources.

Data Name	Data Source	Data Source Site Link
Remote Sensing Image	USGS	http://glovis.usgs.gov/
Yinjiang County Administrative Boundary	State Earth System Science Data Sharing Platform	http://www.geodata.cn/
DEM data	Geospatial Data Cloud site	http://www.gscloud.cn/
Weather data	China Meteorological Data Network	http://data.cma.cn/

### Land surface temperature retrieval

At present, the common algorithms for LST inversion that are based on remote sensing images are radiation conduction equation (atmospheric correction method)^35^, single-channel algorithm^36^, single window algorithm^37^ and split window algorithm^38^. The atmospheric correction method can not only be used to calculate LST with only one thermal infrared band but can also consider surface emissivity and atmospheric radiation effects. Thus, the atmospheric correction method is more comprehensive than the other methods. In addition, three parameters, i.e. transmittance, atmospheric upward radiance brightness and atmospheric downward radiation brightness in this study can be obtained by the atmospheric correction parameter calculator issued by NASA(http://atmcorr.gsfc.nasa.gov), which imports the imaging time, longitude, air pressure of the area and other related information of the input image. It makes the atmospheric correction method easy to use. Therefore, this study used the atmospheric correction method to invert the LST. The concrete method can be found in the “Method Details of LST Retrieval” section in Supplementary Information, in which surface emissivity was calculated by using the NDVI threshold method proposed by Sobrino^39^.

NDVI. We used the significant differences in the red and near-infrared reflectance spectra of green plants to obtain the vegetation index NDVI (Fig. 3). The value of NDVI ranged from −1 to 1. Generally, NDVI > 0 in the growing season indicates vegetation cover. An increase in the NDVI value indicates an increase in green vegetation. NDVI > 0.5 shows good vegetation growth status and large coverage density. The formula for NDVI is expressed in Eq. (1)^26^.

1 $${\rm{NDVI}}=({\rho }_{5}-{\rho }_{4})/({\rho }_{5}+{\rho }_{4}),$$

where, for Landsat 8, *ρ*_4_ is the Band 4 red band (0.64–0.67 µm) reflectance and *ρ*_5_ is the Band 5 near-infrared band (0.85–0.88 µm) reflectance.

### Land use type

**Figure 9:**
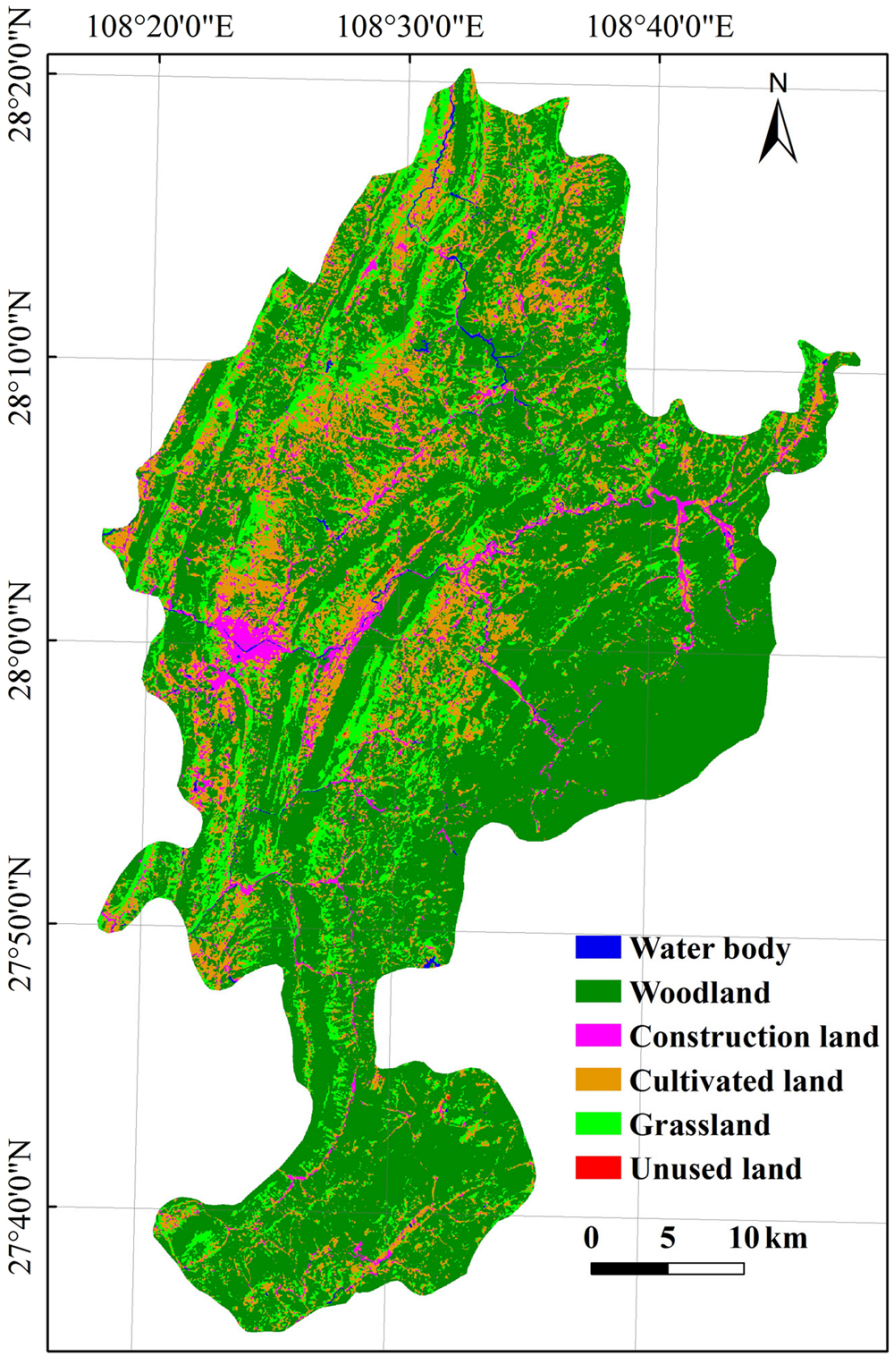
Land use type image of the study area. The image was obtained by ArcGIS 9.3 software (http://www.esri.com).

The August 29, 2016 Landsat 8 image was selected as the data source. CART decision tree^40^ classification was applied to classify the land use types of the study area. This method shows considerable advantages, such as flexibility, intuitiveness, clearness and high efficiency, in remote sensing data classification. The entire study area was divided into six categories, namely, water body, woodland, construction land, cultivated land, grassland and unused land (Fig. 9).

## Electronic supplementary material

Supplementary Information
