# Supplementary material for: Relationship among land surface temperature and LUCC, NDVI in typical karst area
Source: Sci Rep. 2018 Jan 12;8:641. doi: 10.1038/s41598-017-19088-x (PMC5766486; doi:10.1038/s41598-017-19088-x)
Supplement: Supplementary file 1 — Supplementary Information [file 41598_2017_19088_MOESM1_ESM.doc]

# Supplementary Information

**Title: Relationship among Land surface temperature and LUCC, NDVI in typical karst area**

**Authors：**Yuanhong Deng^1, 2, 3^, Shijie Wang^1, 3^, Xiaoyong Bai^1, 3, *^, Yichao Tian^1, 2, 3^, Luhua Wu^1, 2, 4^, Jianyong Xiao^1, 3, 4^, Fei Chen^1, 3, 4^, Qinghuan Qian^1, 3, 4^

^1^State Key Laboratory of Environmental Geochemistry, Institute of Geochemistry, Chinese Academy of Sciences, 99 Lincheng West Road, Guiyang 550081, Guizhou Province, PR China

^2^University of Chinese Academy of Sciences, Beijing 100049, PR China

^3^Puding Karst Ecosystem Observation and Research Station, Chinese Academy of Sciences, Puding 562100, PR China

^4^School of Geography and Environmental Sciences, GuiZhou Normal University, Guiyang 550001, Guizhou Province, PR China

*Corresponding Author: Xiaoyong Bai

Addresses: State Key Laboratory of Environmental Geochemistry, Institute of

Geochemistry, Chinese Academy of Sciences. 99# Lincheng West Road, Guiyang 550081, Guizhou Province, PR China.

E-mail address: baixiaoyong@126.com (X. Bai).

**Supplementary Figures**

**
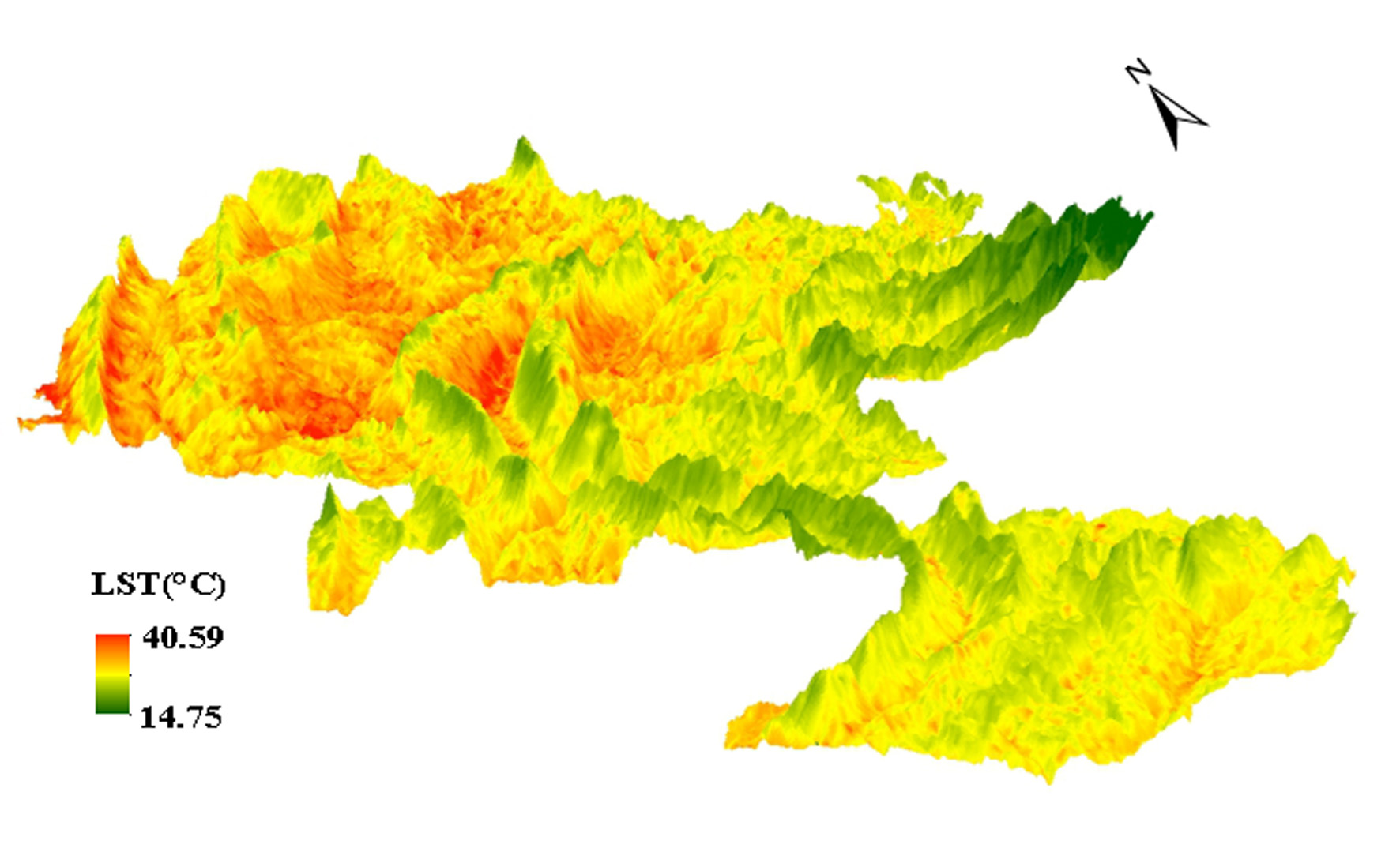
**

**Supplementary Fig. S1 LST 3D map based on DEM.** The image reflects the spatial distribution characteristics of the LST with the terrain change. The LST inversion and DEM elevation maps were used to generate a 3D map by ArcScene, one tool of ArcGIS 9.3 software ([http://www.esri.com](http://www.esri.com/)).


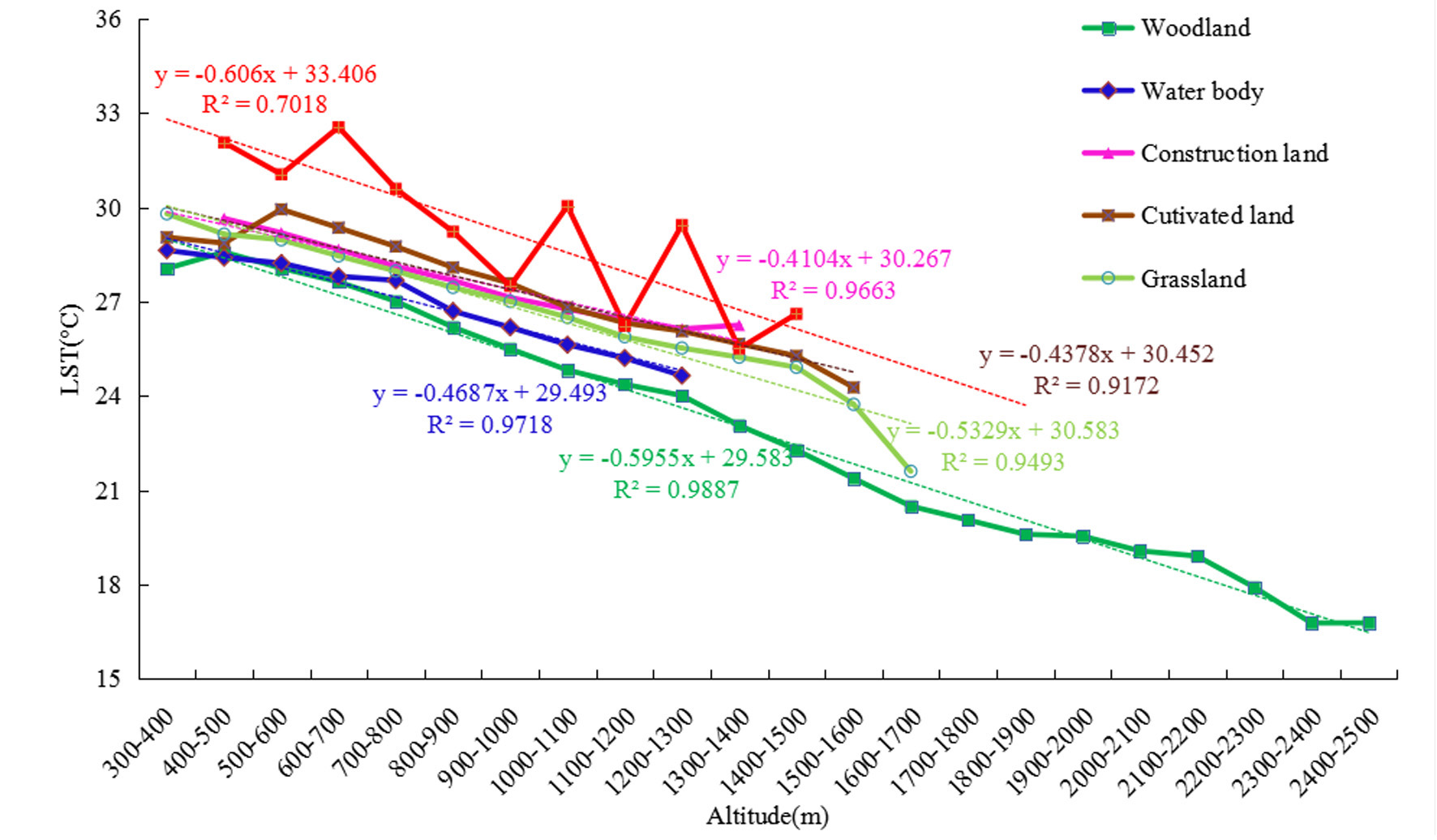


**Supplementary Fig. S2 LST of different land use types at elevations per 100m.** Each solid curve represents that the mean LST of one land use type changes with the elevation increasing; SPSS 22.0 (<https://www.ibm.com/analytics/cn/zh/technology/spss/spss-trials.html>) was used to perform regression analysis on the LST and altitude of each land use type.

**
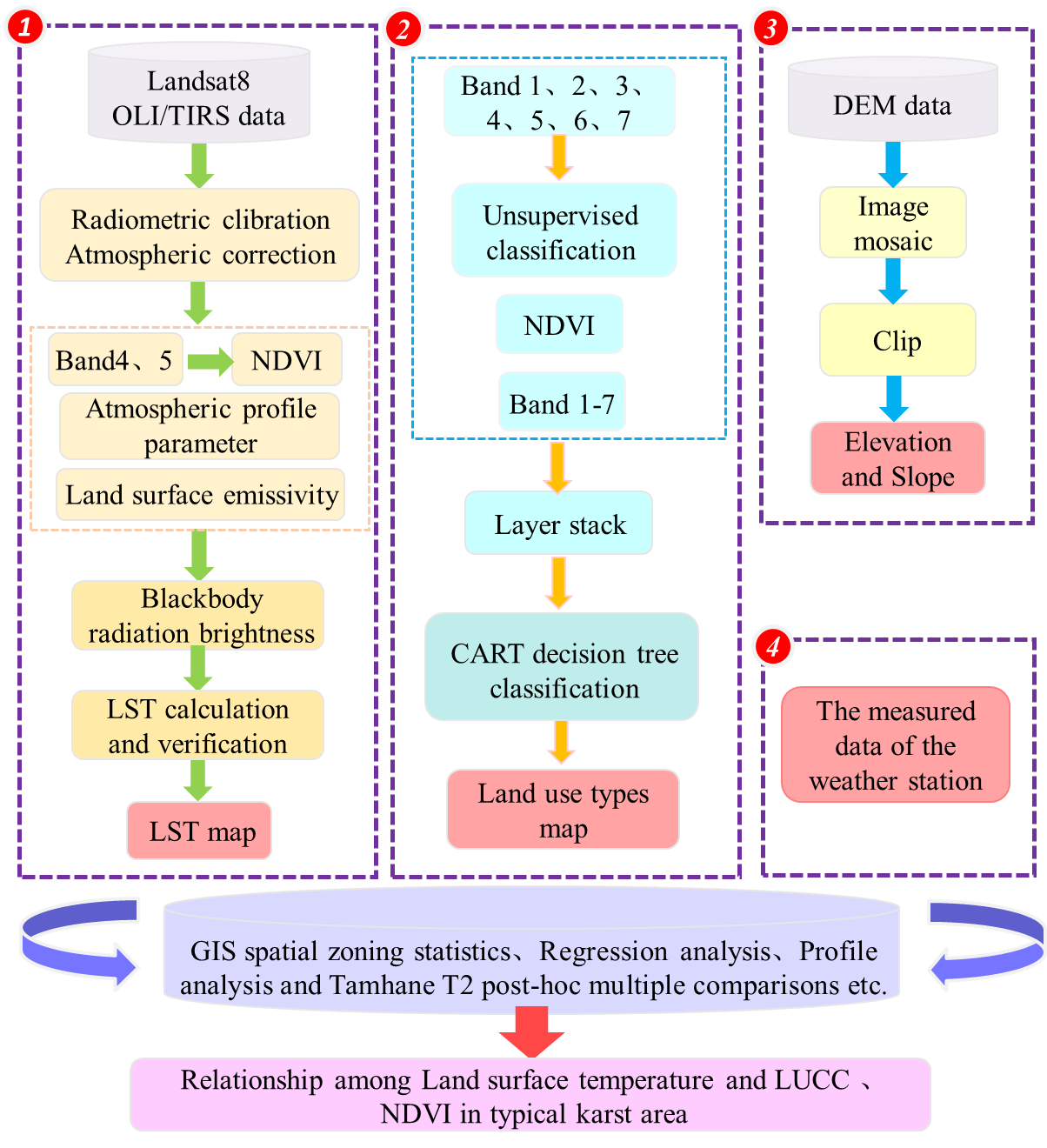
**

**Supplementary Fig. S3 Technical flow chart**

**Supplementary Table**

| Elevation（m） | Water body | Woodland | Construction land | Cultivated lad | Grassland | Unused land |
| --- | --- | --- | --- | --- | --- | --- |
| 400-500 | 0.46 | 1.03 | -0.22 | 1.21 | 0.44 | 1.13 |
| 500-600 | 0.37 | 0.64 | 0.20 | 1.39 | 0.50 | 3.48 |
| 600-700 | -0.11 | 0.69 | 1.14 | 1.53 | 0.66 | 5.14 |
| 700-800 | -0.40 | 0.76 | 0.72 | 1.57 | 0.71 | 3.95 |
| 800-900 | 0.09 | 0.86 | 1.86 | 1.70 | 0.97 | 2.13 |
| 900-1000 | -0.09 | 0.67 | 2.58 | 1.82 | 0.90 | 1.15 |
| 1000-1100 | 0.62 | 0.71 | 0.87 | 1.50 | 1.04 | -2.08 |
| 1100-800 | -0.33 | 0.74 | 2.24 | 1.29 | 0.71 | 1.33 |
| 800-1300 | -0.67 | 0.63 | — | 1.26 | 0.70 | 2.30 |
| 1300-1400 | — | 0.91 | — | 0.89 | 0.51 | 2.60 |
| 1400-1500 | — | 0.86 | — | 0.73 | -0.57 | 0.36 |
| 1500-1600 | — | 0.49 | — | — | — | — |
| 1600-1700 | — | 0.42 | — | — | — | — |
| 1700-1800 | — | 0.67 | — | — | — | — |
| 1800-1900 | — | 0.60 | — | — | — | — |
| 1900-2000 | — | 1.30 | — | — | — | — |
| 2000-2100 | — | 0.88 | — | — | — | — |
| 2100-2200 | — | 0.54 | — | — | — | — |
| 2200-2300 | — | 0.10 | — | — | — | — |
| 2300-2400 | — | 2.75 | — | — | — | — |
| 2400-2500 | — | -0.93 | — | — | — | — |

**Supplementary Table. S1** **the LST mean difference (°C) of different land use types in sunny and shady slope.**

**Method Details of LST Retrieval**

The radiation conduction equation is expressed in Eq. (1).

*L*_λ_ =[*εB*(T_s_) + (1－*ε*) *L*_↓_]τ + *L*_↑_, (1)

where *ε* is the land surface emissivity, *T*_s_ is the true surface temperature (K), *B*(T_s_) is the blackbody radiance, *τ* is transmittance of the thermal infrared band and *L*_↓_ and *L*_↑_ are the atmospheric downward and upward radiance brightness, respectively, which are expressed in W·m^−2^·sr^−1^·µm^−1^.

The Band 10 thermal infrared band of Landsat 8 TIRS and the thermal infrared band of TM/ETM+6 have approximately the same spectral range. Thus, this study used the surface emissivity calculation method similar to TM/ETM+6. Surface emissivity was calculated using the NDVI threshold method proposed by Sobrino^39^. This method considered the following three cases.

(1) When NDVI < 0.2, the entire land was considered bare, and its surface emissivity was the bare land typical emissivity of 0.973.

(2) When NDVI 0.2 ≤ NDVI ≤ 0.5, the pixel was regarded as mixed pixels composed of vegetation and bare soil, and its surface emissivity was calculated by the simplified formula expressed in Eq. (2).

*ε*=0.004*P*_v_ + 0.986, (2)

(3) When NDVI > 0.5, vegetation was considered to be covering the ground completely and its surface emissivity ratio was 0.986. Typical emissivity was denoted as *P*_v_. Vegetation coverage was calculated by the formula expressed in Eq. (3).

*P_v_*=[(NDVI－ NDVI_Soil_)/( NDVI_Veg_－ NDVI_Soil_ )], (3)

Calculation of the blackbody radiant brightness *B* (T_s_): If the surface was Lambertian, then the atmospheric properties of thermal radiation, i.e. blackbody temperature (*T*_s_), which is the same as real surface temperature, could be obtained according to the radiative transfer equation. The formula of radiative transfer equation is expressed in Eq. (4).

*B*(T_s_) =[*L*_λ_ － *L*_↑_－ *τ*(1－*ε*)*L*_↓_] / *τε*,  (4)

In Eq. 4, three parameters, i.e. transmittance *T*_s_, atmospheric upward radiance brightness *L*_↑_ (W·m^−2^·sr^−1^·µm^−1^) and atmospheric downward radiation brightness *L*_↓_ (W·m^−2^·sr^−1^·µm^−1^), can be accessed through the NASA website (http://atmcorr.gsfc.nasa.gov), which imports the imaging time, longitude, air pressure of the area and other related information of the input image. The blackbody brightness *B*(T_s_) unit is W·m^−2^·sr^−1^·µm^−1^.

Calculation of the real surface temperature *T*_s_: After estimating the radiance brightness *B*(T_s_) of the blackbody with the same real surface temperature, the real ground temperature was obtained according to the inverse function of Planck’s law. The formula is expressed in Eq. (5).

*T*_s_=*K*_2_ /ln (*K*_1_ / *B*(T_s_) + 1), (5)

where *K*_1_ and *K*_2_ are calibration constants. For Landsat 8 TIRS Band 10, *K*_1_= 774.89 W/ (m^2^·µm·sr) and *K*_2_ = 1,321.08 K.
